# Correction: Fecal microbiota dysbiosis in macaques and humans within a shared environment

**DOI:** 10.1371/journal.pone.0226092

**Published:** 2019-11-27

**Authors:** Erica T. Grant, Randall C. Kyes, Pensri Kyes, Pauline Trinh, Vickie Ramirez, Tawatchai Tanee, Porntip Pinlaor, Rungtiwa Dangtakot, Peter M. Rabinowitz

The following information is missing from the Competing Interests statement: This journal article was supported by the Grant or Cooperative Agreement Number (5T42OH008433), funded by the Centers for Disease Control and Prevention / National Institute for Occupational Safety and Health. Its contents are solely the responsibility of the authors and do not necessarily represent the official views of the Centers for Disease Control and Prevention or the Department of Health and Human Services. Author PR is the director of the Center for One Health Research and played a role in the study design, data collection and analysis, decision to publish, and preparation of the manuscript. This does not alter our adherence to PLOS ONE policies on sharing data and materials.
