# Supplementary figures and images for: Fecal microbiota dysbiosis in macaques and humans within a shared environment
Source: PLoS One. 2019 May 13;14(5):e0210679. doi: 10.1371/journal.pone.0210679 (PMC6513079; doi:10.1371/journal.pone.0210679)

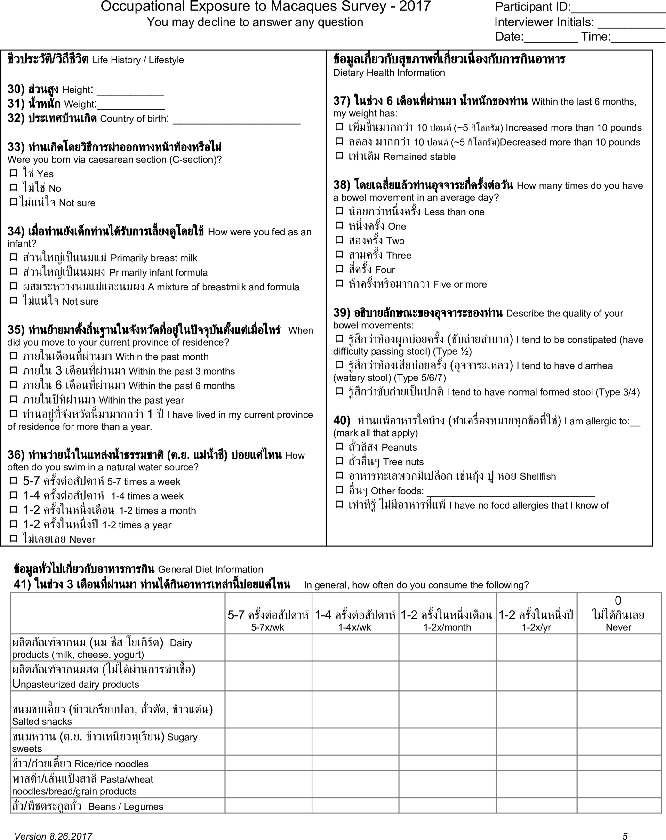

Supplement: S1 File — Questionnaire administered to workers to assess demographic, life history, diet, and general health, in addition to knowledge, attitudes and practices surrounding macaque exposure and zoonoses. Control surveys contained demographic, life history, diet, and general health sections only. (TIF) [file pone.0210679.s001.tif]

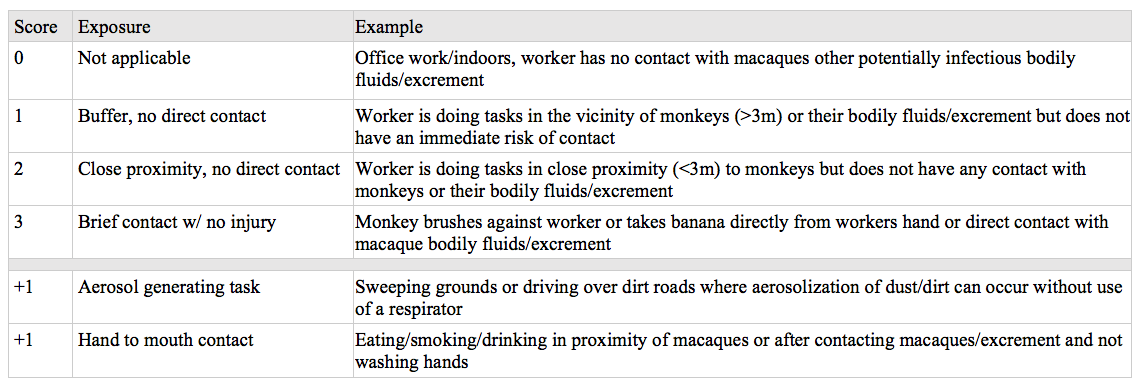

Supplement: S1 Table — Scoring system for video-recorded occupational task observations. Videos were analyzed on the occupational category level, not on an individual level. (TIF) [file pone.0210679.s002.tif]
